# Phylogenomic Insight into *Salinispora* (Bacteria, Actinobacteria) Species Designations

**DOI:** 10.1038/s41598-017-02845-3

**Published:** 2017-06-15

**Authors:** Natalie Millán-Aguiñaga, Krystle L. Chavarria, Juan A. Ugalde, Anne-Catrin Letzel, Greg W. Rouse, Paul R. Jensen

**Affiliations:** 1 Center for Marine Biotechnology and Biomedicine Scripps Institution of Oceanography, University of California San Diego, San Diego, California United States; 2 Universidad Autónoma de Baja California. Facultad de Ciencias Marinas, Ensenada, Baja California Mexico; 3 Centro de Bioinformática y Biología Integrativa, Facultad de Ciencias Biológicas, Universidad Andrés Bella, Santiago, Chile; 4 Marine Biology Research Division Scripps Institution of Oceanography, University of California San Diego, San Diego, California United States

## Abstract

Bacteria represent the most genetically diverse kingdom of life. While great progress has been made in describing this diversity, it remains difficult to identify the phylogenetic and ecological characteristics that delineate groups of bacteria that possess species-like properties. One major challenge associated with species delineations is that not all shared genes have the same evolutionary history, and thus the choice of loci can have a major impact on phylogenetic reconstruction. Sequencing the genomes of large numbers of closely related strains provides new opportunities to distinguish ancestral from acquired alleles and assess the effects of recombination on phylogenetic inference. Here we analyzed the genomes of 119 strains of the marine actinomycete genus *Salinispora*, which is currently comprised of three named species that share 99% 16S rRNA gene sequence identity. While 63% of the core genome showed evidence of recombination, this had no effect on species-level phylogenomic resolution. Recombination did however blur intra-species relationships and biogeographic resolution. The genome-wide average nucleotide identity provided a new perspective on *Salinispora* diversity, revealing as many as seven new species. Patterns of orthologous group distributions reveal a genetic basis to delineation the candidate taxa and insight into the levels of genetic cohesion associated with bacterial species.

## Introduction

The concept that bacteria can be grouped into phylogenetically cohesive clusters with properties that allow them to be regarded as “species” remains controversial^1, 2^. It is challenging to determine which clusters represent species level units of diversity and if ecological or evolutionary theory can be invoked to explain the circumstances that led to their formation^3^. As Gevers *et al*. lament^4^, “any effort to produce a robust species definition is hindered by the lack of a solid theoretical basis explaining the effects of biological processes on cohesion within and divergence between species”. Nonetheless, identifying meaningful groups of bacteria and ascribing formal Latinized names remains useful in clinical, environmental, and experimental contexts^5^. In the absence of a robust species concept for bacteria, we are left with a series of metrics used to gauge the relatedness among strains and phylogenetic frameworks within which species level units of diversity are often arbitrarily assigned.

It is widely recognized that bacterial species concepts should consider both genetic diversity and ecology^2, 6, 7^. Buckley and Roberts stated that, “in moving forward with microbial taxonomy, it is critical to determine whether microorganisms cluster in groups with meaningful commonalities or determine what commonalities may be best used to cluster microorganisms into meaningful groups”^8^. The ecotype model states that bacterial species should fall into well-supported sequence clusters that evolve under cohesive processes and are ecologically distinct and irreversibly separated from each other^6^. A fundamental tenant of this model is that ecologically distinct populations can be recognized as clades in phylogenetic trees and that these clades correspond to fundamental units of diversity or species^2, 6^.

Confounding the common ancestry inferred by phylogenetic reconstruction is homologous recombination. While the efficiency of homologous recombination decreases with increasing genetic distance^9^, it nonetheless occurs between different species^10^. The homologous exchange of genes encoding common housekeeping functions creates challenges for species delineations based on single gene phylogenies and led to the use of techniques such as multi-locus sequence analysis^1^. However, even when multiple loci are considered, an accurate model of vertical inheritance can be difficult to depict due to widespread recombination between species^11, 12^ including ancestral events that have subsequently become fixed among subclades^13^. While the rates of recombination vary widely among bacteria^14^, it remains largely unknown how this process affects species-level phylogenetic resolution when whole genomes are considered.

Whole-genome sequencing has become an indispensable tool for studying genome evolution, genetic diversity, and bacterial species concepts. It has recently been suggested that genome sequences should be used as a source of taxonomic information^15^. One genome-based metric that is gaining acceptance is the Average Nucleotide Identity (ANI) of the sequences shared between strains. It has been shown that an ANI of 95% corresponds to the 70% DNA:DNA hybridization value traditionally used to delineate bacterial species^16^ thus establishing a link to bacterial systematics. Genome sequences also provide unique opportunities to generate highly resolved phylogenies, with automated pipelines to build genomic phylogenies from concatenated protein markers now available^17^. While there is no agreement regarding how many genes it takes to generate a robust phylogenomic evolutionary tree, genome sequences provide comprehensive datasets from which to address evolutionary relationships and predict lateral gene transfer events^18^.

The marine actinomycete genus *Salinispora* provides a valuable model to address bacterial species concepts^19, 20^. It is comprised of three closely related species (*S*. *arenicola*, *S*. *tropica*, and *S*. *pacifica*) within the family Micromonosporeaceae^21, 22^ whose relationships could not be confidently resolved based on 16S rRNA gene phylogeny^23, 24^. The genus is a rich source of structurally diverse natural products^25^, and there is evidence that certain compounds^26^ and their associated biosynthetic gene clusters (BGCs)^27^ are fixed at the species level. This has been used to suggest that secondary metabolites represent ecotype-defining traits for *S*. *tropica* and *S*. *arenicola*. Similar patterns were not observed for *S*. *pacifica* ^26^, the most diverse of the three species^24^. This greater diversity, coupled with the relatively low recombination to mutation rates observed within the *S*. *pacifica* clade, were used to suggest it represents an amalgam of ecotypes or newly diverged species^19^. While all three species are prolific in terms of natural product biosynthesis, it was shown that *S*. *arenicola* differentially invests in interference competition, while *S*. *tropica* invests in growth thus establishing these co-occurring lineages as distinct ecotypes^28^. Here we present a phylogenomic analysis of the genus *Salinispora* based on the shared gene content among 119 strains. The goals were to assess species level diversity and address the effects of recombination on species level phylogenetic reconstruction.

## Results

### General genome characteristics

**Figure 1:**
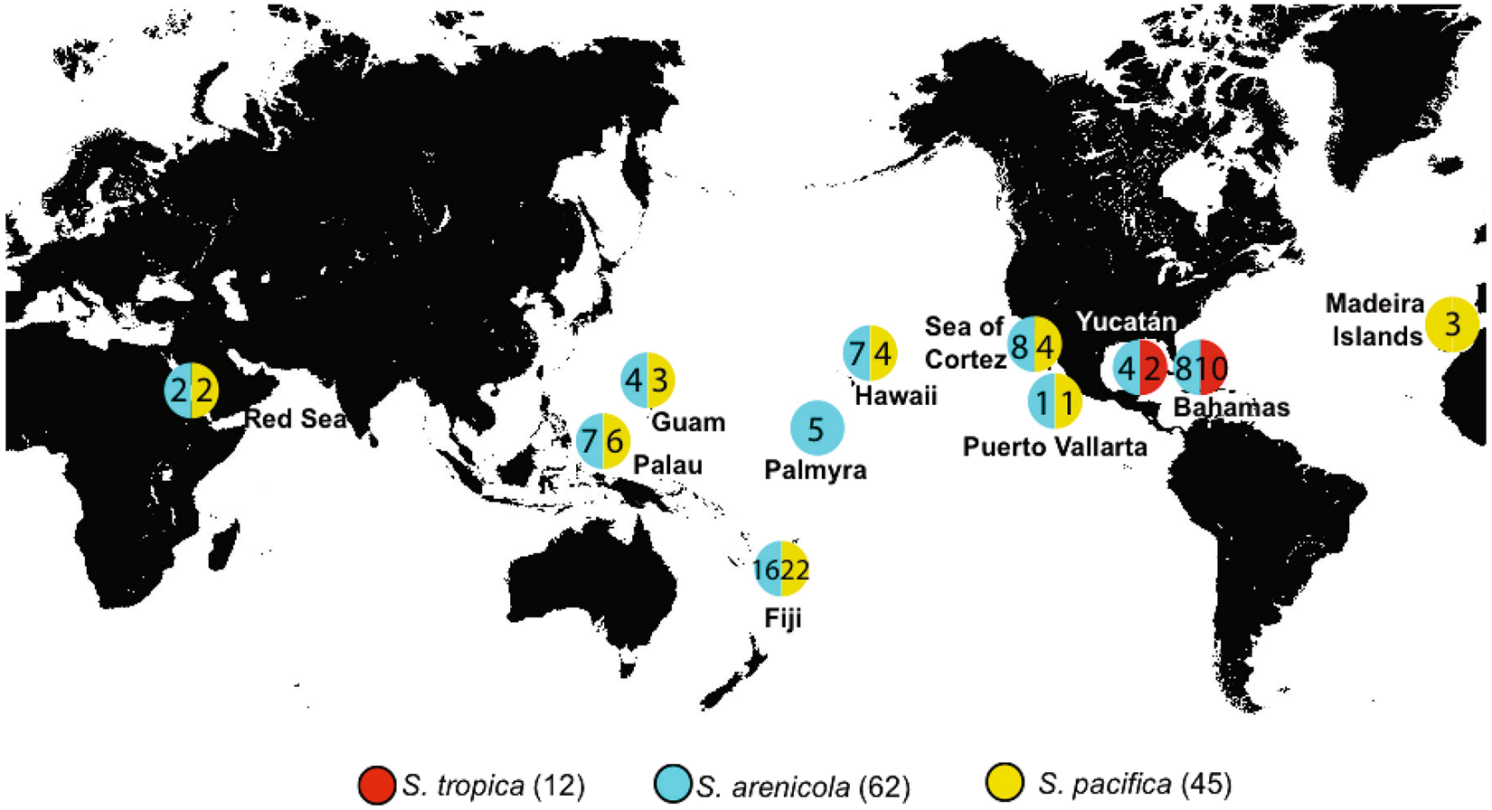
Strain origins. Numbers of strains sequenced at each site for each species with totals in parentheses. Modified with permission from Freel *et al*.^24^, Environ. Microbiol. 14:480–493.

The 119 *Salinispora* genome sequences were derived from 12* S*. *tropica*, 45 *S*. *pacifica*, and 62 *S*. *arenicola* strains isolated from 11 global locations (Fig. 1). All strains were obtained from marine sediment samples collected at depths from 1–700 meters with the exception of four that were derived from marine sponges (Supplementary Table S1). No heterogeneity was observed in the 2–5 copies of the 16S rRNA gene observed in any of the strains. The draft genome sequences averaged 86.3 contigs (Supplementary Table S2) with the majority of sequence data accounted for by a few large contigs in each genome. The average genome size was 5.49 Mb, with the *S*. *arenicola* genomes being larger and containing more genes than the other two species (Table 1).

**Table 1:** Average genome statistics for the genus *Salinispora* and each species.

Taxa	Genome Size (Mbp)	Gene Count	Scaffold Count	GC Content (%)
*Salinispora*	5.57	5148	85	69.7
*S*. *arenicola*	5.74	5234	80	69.8
*S*. *pacifica*	5.42	5079	90	69.9
*S*. *tropica*	5.31	4959	89	69.2

### Orthologous groups

The program FastOrtho was used to predict a total of 13,512 orthologous groups (OGs) and 4,980 single copy genes (singletons) among the 119 *Salinispora* genomes revealing a pan-genome that totaled 18,492 protein families. The core genome consists of 2603 OGs shared by all 119 strains, with 2362 of these occurring as a single-copy in each strain. The core genome represents 51% of the average gene content across the genus. Based on the annotation or putative function of the OGs, more than 50% of the pan-genome is comprised of poorly characterized genes (Supplementary Fig. S1). As observed in other genera^29^, the core genome is enriched in functionally annotated genes with the largest group (35%) attributed to metabolism. Similar analyses performed at the species level reveal that *S*. *tropica* has the largest core genome representing 78.68% of the average gene content. Conversely, *S*. *pacifica* displays the smallest core genome at 56.10% of the average gene content while *S*. *arenicola* was intermediate at 67.42%. As expected, the core genomes vary inversely as a function of the diversity of the strains sequenced within each species.

**Figure 2:**
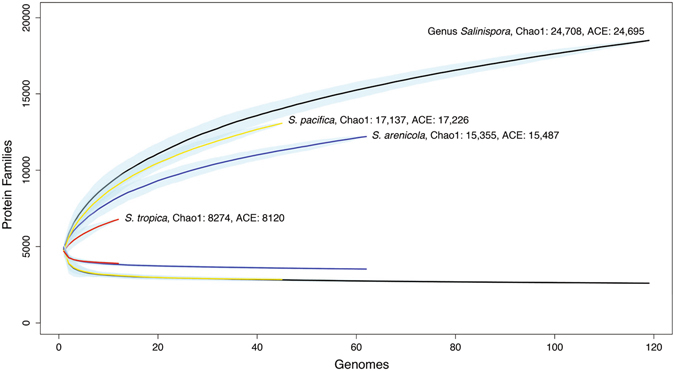
Rarefaction curves. Orthologous groups (protein families) plotted vs. the number of sequenced genomes. Core genomes (lower curves) and pan-genomes (upper curves) are shown for the genus and each species. Black: genus, red: *S*. *tropica*, blue: *S*. *arenicola*, yellow: *S*. *pacifica*. Blue shading indicates standard error. Diversity estimates using Chao1 and ACE are given.

Rarefaction curves were computed to estimate how effectively gene content had been sampled (Fig. 2). There is clear evidence for saturation when the genus or species-level core genomes are considered and thus the common genetic features that characterize the cultured representatives of these taxa have largely been identified. It is notable that the curves generated from the *S*. *tropica* and *S*. *arenicola* core genomes are largely identical, while the curve for *S*. *pacifica* resembles that describing the genus. For the pan-genomes however, it can be predicted that additional sequencing will reveal additional genetic diversity at all levels. Diversity estimators (Chao1 and ACE) predict more than 24,000 protein families at the genus-level relative to the 18,492 observed. Of the three species, *S*. *pacifica* shows the highest observed and predicted genetic diversity.

### Effects of recombination on *Salinispora* phylogeny

**Figure 3:**
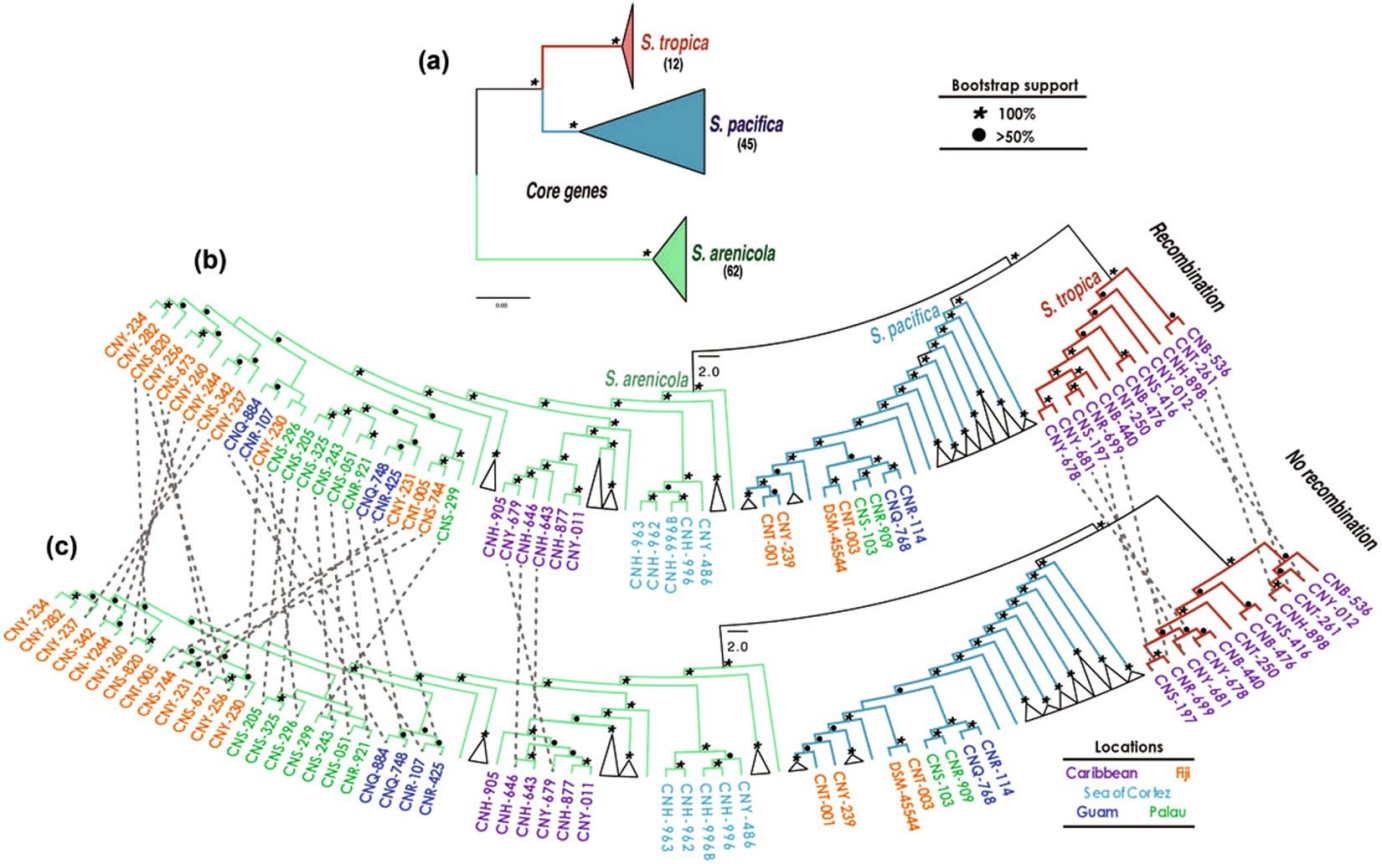
*Salinispora* maximum likelihood phylogeny. (**a**) Collapsed phylogenomic tree based on a concatenation of 2362 shared, single-copy genes. Number of strains analyzed for each species is shown in parentheses. Non-collapsed trees are presented in Fig. 5. (**b)** Phylogeny based on genes with evidence of recombination. (**c)** Phylogeny based on genes with no evidence of recombination. Strain numbers are given in cases where the tree topologies differ. When possible, branches with the same topology in both trees were collapsed. Dashed lines depict positional changes of strains in the trees. Branches are color-coded by species. Symbols on the branches represent the support from 1,000 bootstrap replicates. Strain numbers are color-coded by location.

The 2362 single copy genes identified in the core *Salinispora* genome (hereafter referred to as the single copy core or SCC) were used to generate a concatenated phylogeny that clearly resolved the genus into three well supported clades in accordance with prior species-level relationships (Fig. 3)^19^. This phylogeny supports the relatively high level of diversity reported for *S*. *pacifica*. We next used the program PhiPack to address the effects of recombination on phylogenetic reconstruction^30^. This led to the detection of 1,486 SCC genes (62.9%) with evidence of recombination. The remaining 876 genes had no evidence of recombination and are considered the “minimum” core genome. We generated a second concatenated phylogenomic tree using the minimum core genome (Fig. 3) and manually compared this to the individual gene trees for each of the 1,486 SCC genes with evidence of recombination. We identified 635 genes (42.7% of those under recombination and 26.9% of the SCC) that displayed incongruent species level phylogenies for at least one strain relative to the concatenated phylogenomic tree (Supplementary Fig. S2). To test for the aggregate effects of recombination, a third concatenated phylogeny was generated using the 1486 SCC genes with evidence of recombination (Fig. 3). Surprisingly, all three trees were both congruent and similarly well supported in terms of the three major clades associated with the named *Salinispora* species. Thus, recombination did not affect *Salinispora* species-level phylogenomic resolution. The large numbers of genes that displayed incongruent species-level phylogenies were insufficient to affect interspecies relationships when taken in the context of the larger gene pools. Notably, the tree generated from the minimum core genome reveals clear biogeographic patterns within *S*. *arenicola* that were obscured when genes subject to recombination were included (Fig. 3).

These phylogenies were based on the concatenation of various gene sets into a single multiple alignment and the estimation of a single tree from this super-alignment. Given that alternative phylogenetic methods can infer different relationships, the data were re-analyzed using ASTRAL (Accurate Species TRee ALgorithm), a coalescent-based method to summarize individual gene trees into a single species tree^31^. ASTRAL identifies the species tree that agrees with the largest number of individual trees and can be more accurate than maximum likelihood analyses when using a concatenated gene set^32^. Given this, we performed a similar set of analyses using ASTRAL, which resulted in trees that were congruent at the species level with the concatenated trees (Supplementary Figs S3 and S4), thus providing further support for these phylogenetic patterns.

### ANI and ANI-AF metrics

**Figure 4:**
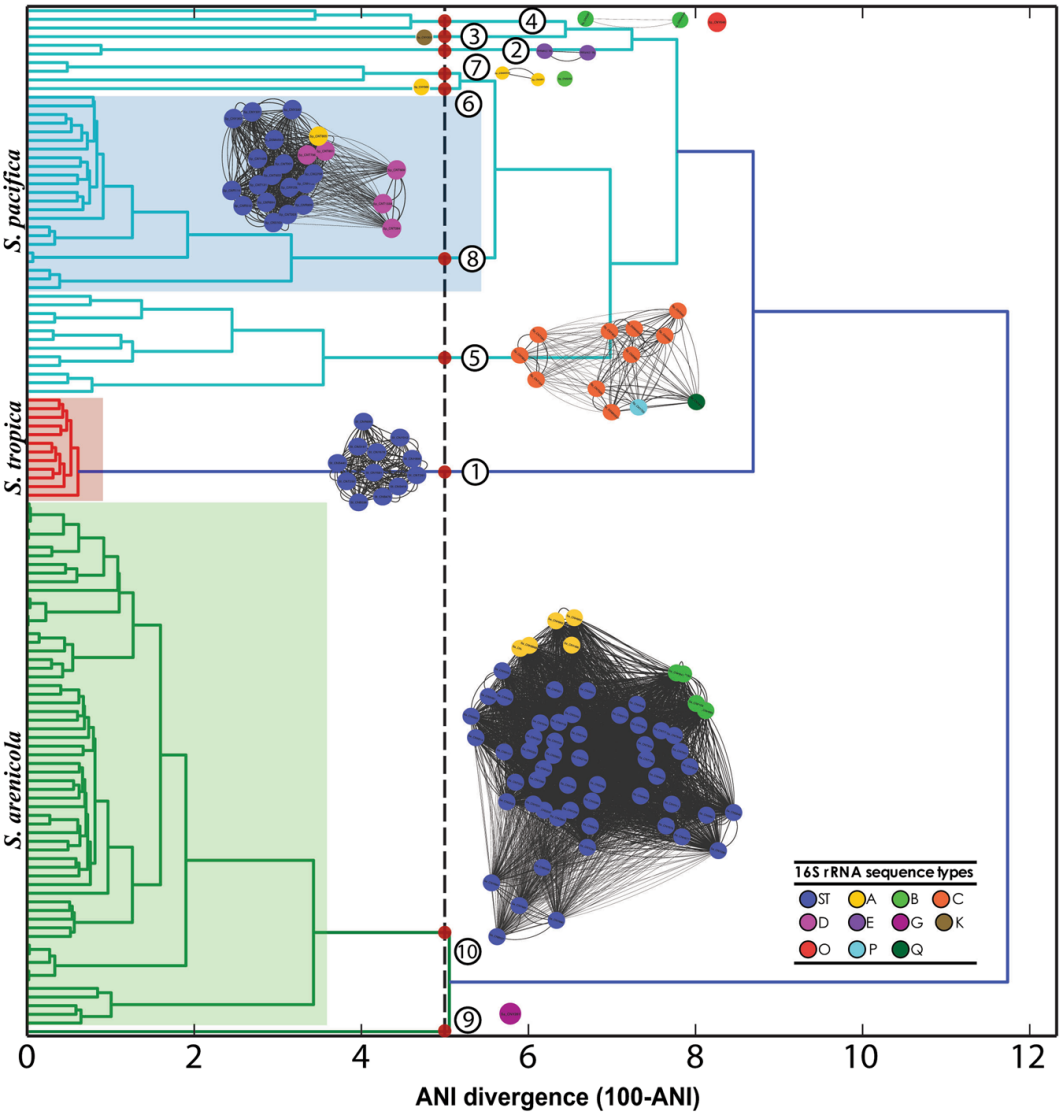
Average Nucleotide Identity (ANI) distance matrix. The vertical dashed line represents 95% ANI. Strains associated with the three primary clades are delineated by green (*S*. *arenicola*), red (*S*. *tropica*), and light blue (*S*. *pacifica*) branches. ANI clades that share >95% and are associated with type strains are shaded. Red circles and corresponding numbers represent all lineages that share <95% ANI values including seven (2–7, 9) that do not contain type strains. ANI-AF networks are shown adjacent to the corresponding regions in the dendogram. Each node represents a strain and is color-coded based on the 16S rRNA gene sequence types (single nucleotide polymorphisms) observed for each species.

**Figure 5:**
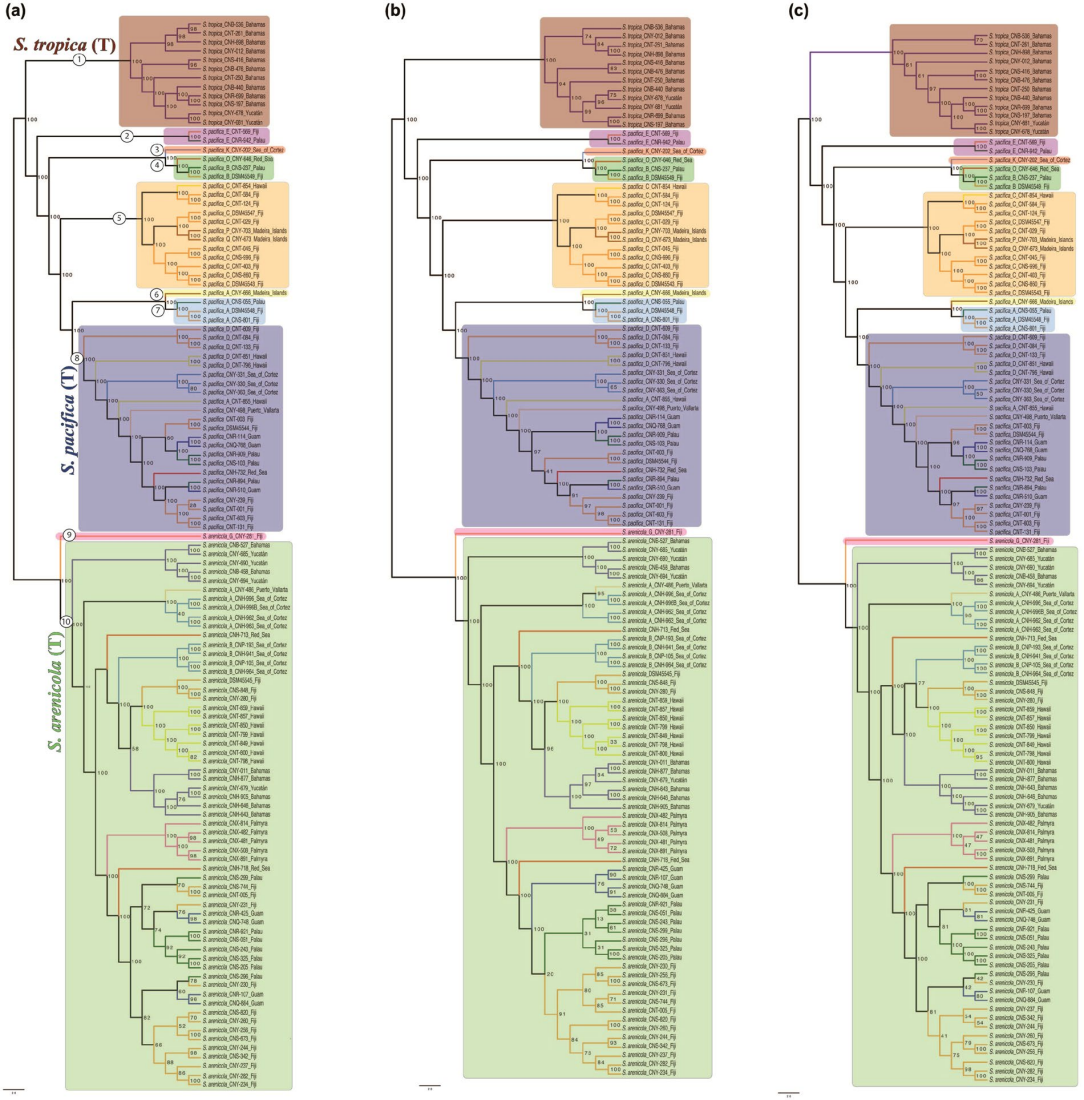
Effects of recombination on phylogenetic resolution using ANI species designations. (**a)** Phylogenomic tree based on a concatenation of 2362 shared, single-copy genes. Each sequence or clade that shares <95% ANI with neighboring strains is numbered 1–10 (corresponding to Fig. 4) and shaded with a different color. Species names are listed corresponding to the clades associated with the type strains (T). (**b)** Phylogeny based on genes with no evidence of recombination. (**c)** Phylogeny based on genes with evidence of recombination.

We next asked if the species assignments inferred from the three primary clades observed in the SCC phylogenomic tree, which have been used to distinguish among the three *Salinispora* species^24^, were in accordance with the proposal that ANI values between members of the same species should be ≥95%^16^. A distance matrix based on ANI values reveals a dendrogram with three primary bifurcations that are congruent with the phylogenomic tree (Fig. 4). However, many strains within the three primary clades fall below the 95% ANI metric, suggesting the existence of additional species-level diversity. More specifically, seven branches within the primary *S*. *pacifica* lineage could be considered distinct species based on this metric. The most populated branch includes the *S*. *pacifica* type strain (CNR-114)^22^ and 22 additional strains isolated from seven of the global collection sites. The second most populated branch includes 12 strains recovered largely from Fiji while the remaining five branches include one to three strains. The strains comprising these seven lineages are clearly resolved in the expanded phylogenomic tree (Fig. 5) and suggest that the primary clade sister to *S*. *tropica* is comprised of as many as seven distinct species of which *S*. *pacifica* is one. Similarly, the *S*. *arenicola* clade includes two branches that fall below the 95% ANI level. These consist of the single strain CNY-281 and a second branch that contains all of the remaining *S*. *arenicola* strains including the type strain. Conversely, the *S*. *tropica* clade is represented by a single branch within which all strains share >95% ANI. Thus, according to the ANI analyses, the 119 *Salinispora* strains represent as many as 10 different species.

We analyzed the data further using the ANI-AF method^33^, which considers only coding orthologous groups (CDS: From Coding DNA Sequences) and the alignment fraction (AF) between genomes as a measure of relatedness. The values suggested to delineate species are ANI >96.5 and AF >0.6. The ANI-AF results for *S*. *tropica* and *S*. *arenicola* remain the same, however within the *S*. *pacifica* clade, CNS-055 and CNY-646 are delineated as two additional species. Based on the ANI species designations, we re-investigated the effects of recombination on species-level phylogenomic resolution and once again found no effect (Fig. 5). The 10 candidate *Salinispora* species are all clearly resolved both from their minimum core genomes and the SCC genes with evidence of recombination. Thus, recombination does not affect the phylogenetic resolution of the major lineages associated with the three currently named *Salinispora* species or the ten candidate species into which these lineages could be delineated based on ANI.

**Figure 6:**
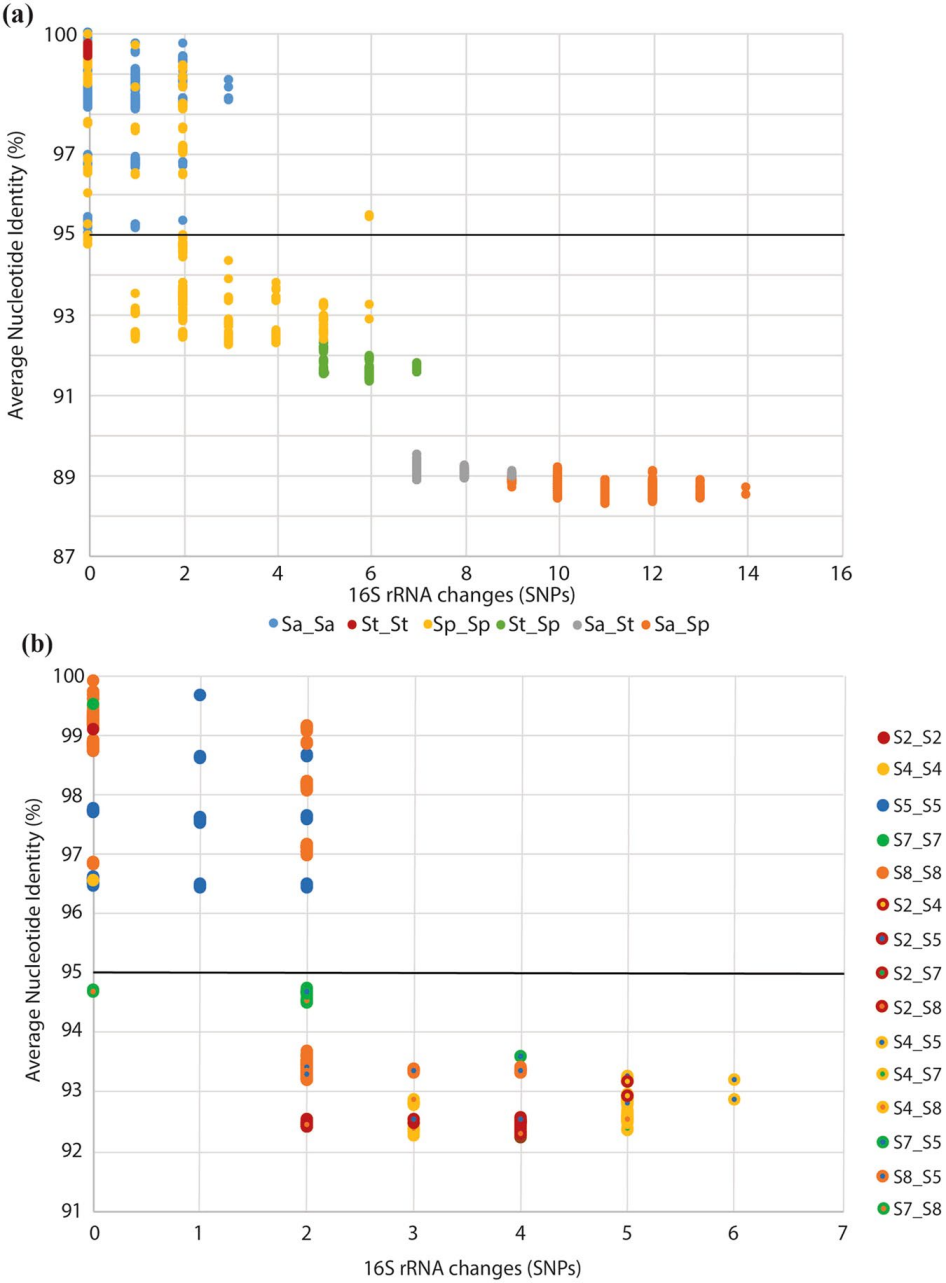
Relationships between 16S rRNA diversity and Average Nucleotide Identity (ANI). The black line indicates 95% ANI. (**a**) Inter- and intraclade comparisons among the three major lineages represented by *S*. *tropica* (St), *S*. *arenicola* (Sa), *S*. *pacifica* (Sp). (**b**) Inter- and intraclade comparisons among the *S*. *pacifica* clades (2–8) as identified in Figs 4 and 5.

*Salinispora* 16S rRNA sequence types (single nucleotide polymorphisms) correspond surprisingly well to the ANI-AF clustering (Fig. 4). To further explore these relationships, we plotted 16S rRNA sequence divergence vs. ANI (Fig. 6). Interspecies comparisons based on the three primary clades in the *Salinispora* phylogeny revealed from five (St-Sp) to 14 (Sa-Sp) changes in the 16S rRNA gene. All *S*. *arenicola* and *S*. *tropica* intra-species comparisons are above 95% ANI and reveal at most three 16S polymorphisms while many of the *S*. *pacifica* intraspecies comparisons fall below this line and include up to six SNPs. A linear regression of the data and best-fit line reveals that a 95% ANI value corresponds to 3.1 changes in the 16S rRNA gene (Supplementary Fig. S5). Given that many of the intra-clade comparisons for the major clade that includes *S*. *pacifica* fall below 95% ANI, we performed a separate analysis of these seven lineages (Fig. 6). As expected, all comparisons within these seven clades fall above and all between clade comparisons fall below 95% ANI. In this case however, the inter-clade comparisons differ from 0–6 16S rRNA SNPs.

### Genetic basis for species delineations

We previously reported species-specific patterns of secondary metabolite production in *S*. *arenicola* and *S*. *tropica* ^26^, however similar patterns were not observed for *S*. *pacifica* ^25^. To further explore this concept in *S*. *pacifica*, we identified biosynthetic gene clusters (BGCs) associated with secondary metabolism using antiSMASH^34^ and manual annotations. We then prepared a similarity matrix using the presence/absence of BGCs in each strain as input (Supplementary Fig. S6). Except for the position of CNY-666, the BGC dendrogram and the phylogenomic tree are largely identical. To further test for evidence of genetic or functional traits that differentiate the candidate *Salinispora* species, we performed similar analyses based on the presence or absence of orthologous groups associated with 23 COG categories (Supplementary Table S3) and found that categories C (energy production and conversion, Supplementary Fig. S7), E (amino acid transport and metabolism), G (carbohydrate transport and metabolism), H (coenzyme transport and metabolism), I (lipid transport and metabolism), and R (general function prediction) consistently delineated the candidate species within the primary *S*. *pacifica* lineage in accordance with the phylogenomic tree (Fig. 5). Thus, in addition to secondary metabolism, there appear to be major genetic differences among the candidate *S*. *pacifica* species.

**Figure 7:**
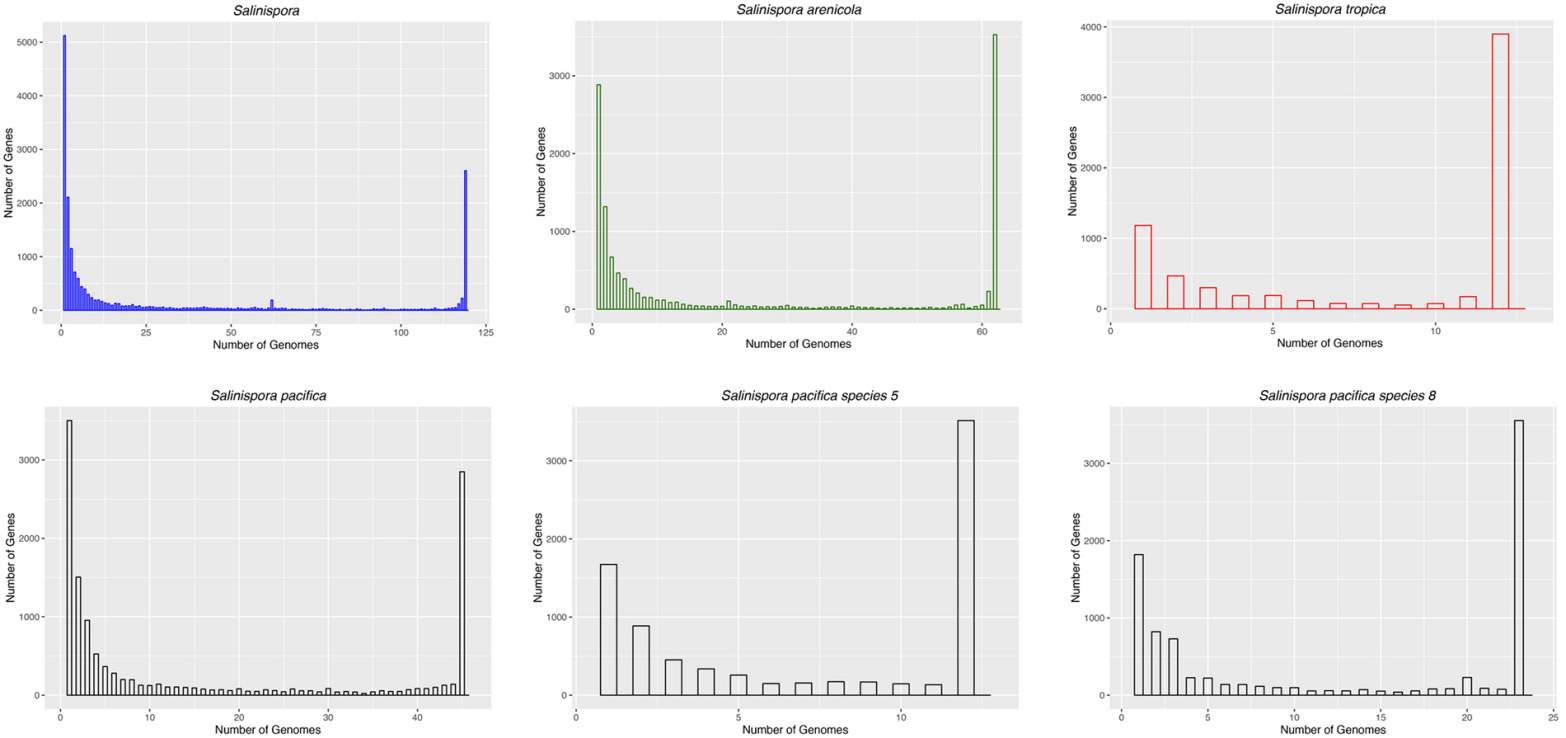
Numbers of orthologous groups found across all genomes (upper left), *S*. *arenicola* genomes (upper center), *S*. *tropica* genomes (upper right), *S*. *pacifica* genomes (bottom left), *S*. *pacifica* candidate species*“*Sp1” (bottom center), *S*. *pacifica* candidate species “Sp2” (bottom right). The histograms were generated from the pan-genomes excluding singletons and recent paralogs.

While differences in gene content provide one mechanism to distinguish among species, it can also be expected that the same species will share a certain level of genetic homogeneity. To explore these concepts, we plotted OG distributions across various taxonomic levels (Fig. 7). All histograms clearly show that most genes are either rare or occur in all strains. When the genus is assessed, the core genome represents only 14% of the pan-genome and the relatively large spike in the left portion of the graph provides little evidence for genetic cohesion, as might be expected from a genus comprised of multiple species^29^. Conversely, when *S*. *arenicola* and *S*. *tropica* are plotted, the core genomes represent 29% and 58% of the respective pan-genomes, and the numbers of OGs observed in all strains exceed those observed in only one strain. In the primary *S*. *pacifica* lineage however, the pattern is similar to that detected for the genus, with the core genome representing only 22% of the pan-genome. As was observed in the rarefaction curves, these results are more similar to those for the genus than for either *S*. *tropica* or *S*. *arenicola*. We performed similar analyses using the two most populated candidate species within the primary *S*. *pacifica* lineage and observed OG distributions that resemble *S*. *tropica* and *S*. *arenicola*, with core genomes between 40% and 44% of the pan-genomes. These patterns may provide added insight into the levels of genetic cohesion expected for a bacterial species.

## Discussion

The comparison of large numbers of genome sequences derived from closely related bacteria provides a unique opportunity to address bacterial species concepts and the metrics commonly employed to assess sequence-based relationships. Fundamental to this process is the identification of the core genome, which defines the common genomic features that characterize the strains under consideration. As can be expected, core genomes vary widely depending on the diversity of strains and number of genomes examined^35–38^. Nonetheless, this shared gene pool provides unparalleled opportunities to assess levels of sequence divergence and generate comprehensive molecular phylogenies that can be used to infer evolutionary relationships and identify alleles that have been exchanged by homologous recombination.

Homologous recombination provides a mechanism to repair damaged DNA and generate genetic diversity within bacterial genomes^39^. While molecular phylogeny is the primary tool used to assess bacterial diversity, it is well documented that homologous recombination blurs species boundaries and can prevent accurate species delineations^40^. By analyzing the single copy core (SCC) genome associated with 119 closely related *Salinispora* strains, it was possible to generate a detailed and highly supported phylogeny that revealed three primary lineages in agreement with previously established relationships among the three currently names species^19^. Although 63% of the SCC showed evidence of recombination for at least one strain, this had no effect on the evolutionary relationships among the three primary clades. However, removing loci that showed evidence of recombination from the analyses revealed enhanced biogeographic patterning within the *S*. *arenicola* clade and new evidence for endemism among the structured populations. A majority of genes that displayed evidence of recombination generated phylogenies that were congruent with the established species phylogeny, indicating that most of these events occurred within the three primary lineages as opposed to between them. This is in agreement with the concept that recombination provides a cohesive force that maintains species level units of diversity^41^. However, the large number of core genes that generated incongruent species phylogenies (27%) reveals the importance of selecting the appropriate phylogenetic markers and the power of phylogenomics to overcome this potential source of misleading phylogenetic inference.

ANI analyses revealed that the three primary *Salinispora* clades could be further delineated into as many as 10 different species, all of which could be confidently resolved even when recombinant alleles were included. While three of these lineages are associated with named species^21, 22^, six belong to the relatively diverse clade that is sister to *S*. *tropica* and contains the *S*. *pacifica* type strain. This supports the previous suggestion that this clade represents an amalgam of ecotypes or newly diverges species based on its relative low rates of recombination to mutation^19^. The possibility that 10 species are represented among a group of strains that share 99% 16S sequence identity supports the concept that this conserved phylogenetic marker is not the best choice for species-level resolution^4^. Nonetheless, 95% ANI corresponded to approximately three changes in the 16S gene thus indicating that any change in this conserved marker may be meaningful from a taxonomic perspective.

The phylogenetic resolution achieved here is in stark contrast to the genus *Streptomyces*, where high recombination to mutation rates detected using MLSA approaches led to the suggestion that phylogenetic relationships within this genus were better represented by a reticulate network^12^. It remains unclear why the effects of recombination on phylogenetic resolution differ between two taxa within the same bacterial order, however it may relate to the diversity of the strains examined and the number of alleles assessed in the different studies. Furthermore, it is interesting to speculate that among *Streptomyces* spp., the acquisition of alleles resistant to the many antibiotics they produce may contribute to the high levels of homologous recombination observed, as was shown for the *rpoB* phylogeny in *Salinispora* spp.^19^ and exploited to identify the biological targets of secondary metabolites prior to their discovery^42^.

In support of this concept, natural product BGCs are frequently exchanged by horizontal gene transfer^27^ and often include a resistant version of the target on which the encoded compounds act^43^. These resistance genes often have homologs in the core genome and can appear as a second copy of a housekeeping gene^44^. In other cases, the resistant housekeeping gene associated with the BGC is the only copy in the genome^19^, suggesting the ancestral allele was subsequently lost. These later events are difficult to distinguish from homologous recombination and may account for some of the single copy genes identified as under recombination in this study. Thus, the ability to produce and be resistant to secondary metabolites may represent a major factor confounding phylogenetic resolution among bacteria enriched in this metabolic capacity. Nonetheless, phylogenomic approaches were sufficient to overcome these incongruences, leading to the generation of stable trees with highly supported clades that can be further evaluated for species-like properties.

Linking strains that can be delineated based on phylogeny or sequence similarity with distinct ecological traits remains a critical and challenging component of microbial ecology. In this regard, it was possible to show that the distributions of secondary metabolite BGCs and six COG categories were largely congruent with the 10 candidate *Salinispora* species delineated based on ANI and resolved in the phylogenomic tree. Thus, there appears to be considerable genetic cohesion among these lineages including within﻿ the category of secondary metabolism, which has been reported to represent an important species defining trait for this genus^45^. Ultimately, resolving the genetic and ecological differences among these closely related groups of bacteria, as initially demonstrated between strains of *S*. *tropica* and *S*. *arenicola* ^28^, will be an essential component of testing the hypothesis that they maintain the properties expected of different species. While it remains to be determined if these results apply more broadly to other groups of bacteria, the expansive growth of genome sequence data will provide ample opportunities to explore species concepts in the future.

## Methods

### Genome sequencing

Genome sequencing was conducted by the U.S. Department of Energy Joint Genome Institute as part of the Community Science Program (http://jgi.doe.gov/user-program-info/community-science-program/). DNA was extracted and the sequence annotation and assembly carried out as previously described^27^. Genomic data is available from the Integrated Microbial Genomes (IMG) database (https://img.jgi.doe.gov). IMG genomes ID and NCBI taxon numbers are provided in Supplementary Table S1.

### Orthologous group computation

A total of 119 *Salinispora* strains (12*S*. *tropica*, 62*S*. *arenicola* and 45*S*. *pacifica*) from 11 different locations (Fig. 1, Supplementary Table S1) were analyzed using the program FastOrtho^46^ to identify groups of orthologous protein coding genes (orthologous groups, OGs). This program is a reimplementation of OrthoMCL^47^ and performs a bidirectional best blast amino-acid analysis. Clustering based on a percent match was performed using default parameters (cutoff = 70, e-value cutoff = 1e^−05^, and inflation index (I) = 1.5) (https://github.com/juanu/MicroCompGenomics). Rarefaction curves and diversity estimates were generated using the vegan package in R (http://www.R-project.org). The output matrix of FastOrtho was processed to identify species-specific orthologous groups using an Excel macro (https://github.com/joseluisrc/FindSharedGenes). Histograms were plotted from the presence-absence matrix of OGs using the qplot function and the ggplot2 package in R (http://www.R-project.org).

### Identification of the core genome and the detection of recombination

A series of custom python scripts (htps://github.com/juanu/MicroCompGenomics) were applied to the FastOrtho results to identify the OG members that included gene duplications (paralogs). Orthologous groups that included paralogs were removed to generate the single copy core (SCC) gene pool. The nucleotide sequences of the individual SCC genes in each strain were aligned using MUSCLE with default parameters and trimmed for quality using GBlocks. The SCC genes were screened for evidence of recombination using PhiPack^48^, which included the statistical tests PHI, MaxChi, and Neighbor Similarity Score, all with default parameters. Recombination was inferred when p-values less than 0.01 were detected. Attempts to use the Recombination Detection Program^49^ failed due to the large number of loci examined.

### Phylogenetic analyses

A maximum likelihood (ML) tree was generated for each SCC gene using the program RAxML (command line version) with mid-point rooting and 100 bootstraps (Stamatakis, 2006). The individual gene trees were visualized using the program FigTree v1.3.1 (http://tree.bio.ed.ac.uk/software/figtree). Trimmed alignments of each gene were then concatenated and used to build ML phylogenies using RAxML^50^ implemented on the CIPRES portal v2.2 at the San Diego Supercomputer Center^51^. Analyses included 1,000 bootstrap replicates using the most complex model (GTR + GAMMA) for both bootstrapping and final ML optimization using default parameter settings. The resulting tree was rooted at the mid-point and visualized using FigTree. Individual SCC gene trees that showed incongruence at the species level with the concatenated tree were scored as under recombination. Two additional concatenated SCC gene trees were then generated for the subsets of this gene pool that included only genes with evidence of recombination and only genes with no evidence of recombination using the methods described above. A similar set of SCC species trees was also generated using the program ASTRAL^31^, which uses the best RAxML trees for each gene tree.

### Average nucleotide identity and alignment fraction

The average nucleotide identity (ANI) and alignment fraction (AF) were determined for all 119 *Salinispora* genomes using published methods^16, 33^. ANI values were calculated for all pairwise comparisons and used to compile a distance matrix representing ANI divergence (100 - ANI). The custom scripts used to perform these analyses and generate the ANI dendrogram are available (https://github.com/juanu/ANI_analysis/blob/master/ANI_blastn.py and https://ani.jgi-psf.org/html/download.php). Cytoscape 3.3.0 was used to visualize the results^52^.

### Clustering based on COG category and functional traits

The OGs were classified into five major functional categories based on the FastOrtho results and further divided into clusters of orthologous groups (COGs, Supplementary Table S3). These classifications were used to build hierarchical cluster analyses based on the presence/absence of OGs assigned to each COG category using the function hclust and the method “average” in the R package (http://www.R-project.org). A hierarchical cluster analysis was similarly generated using the presence/absence of secondary metabolite BGCs predicted for the 119 *Salinispora* genomes using antiSMASH^34^ as previously described^27^.

## Electronic supplementary material

Supplementary Information
