# Supplementary material for: Phylogenomic Insight into Salinispora (Bacteria, Actinobacteria) Species Designations
Source: Sci Rep. 2017 Jun 15;7:3564. doi: 10.1038/s41598-017-02845-3 (PMC5472633; doi:10.1038/s41598-017-02845-3)
Supplement: Supplementary file 1 — Supplementary Information [file 41598_2017_2845_MOESM1_ESM.docx]

**Supplementary Information**

Phylogenomic Insight into *Salinispora* (Bacteria, Actinobacteria) Species Designations

Natalie Millán-Aguiñaga, Krystle L. Chavarria, Juan A. Ugalde, Anne-Catrin Letzel, Greg W. Rouse, Paul R. Jensen

**Supplementary Table S1.** General characteristics of the 119 *Salinispora* strains.

**Supplementary Table S2.** Genome information.


**Supplementary Table S3.** Major functional categories and Clusters of Orthologous Groups (COGs, bold letters) to which the *Salinispora* orthologous groups were assigned.

| Cellular processes and signaling  (CP&S) | **[D]** Cell cycle control, cell division, chromosome partitioning  **[M]** Cell wall, membrane, envelope biogenesis  **[N]** Cell motility  **[O]** Post-translational, modification, protein turnover, and chaperones  **[T]** Signal transduction mechanisms  **[U]** Intracellular trafficking, secretion, and vesicular transport  **[V]** Defense mechanism  **[Y]** Nuclear structure |
| --- | --- |
| Information storage and processing  (IS&P) | **[A]** RNA processing and modification  **[B]** Chromatin structure and dynamics  **[J]** Translation, ribosomal structure and biogenesis  **[K]** Transcription  **[L]** Replication, recombination and repair |
| Metabolism  (M) | **[C]** Energy production and conversion  **[E]** Amino acid transport and metabolism **[F]** Nucleotide transport and metabolism **[G]** Carbohydrate transport and metabolism  **[H]** Coenzyme transport and metabolism  **[I]** Lipid transport and metabolism  **[P]** Inorganic ion transport and metabolism |
| Secondary metabolism  (SM) | **[Q]** Secondary metabolites biosynthesis, transport and catabolism |
| Poorly characterized  (PC) | **[R]** General function prediction only  **[S]** Function unknown |


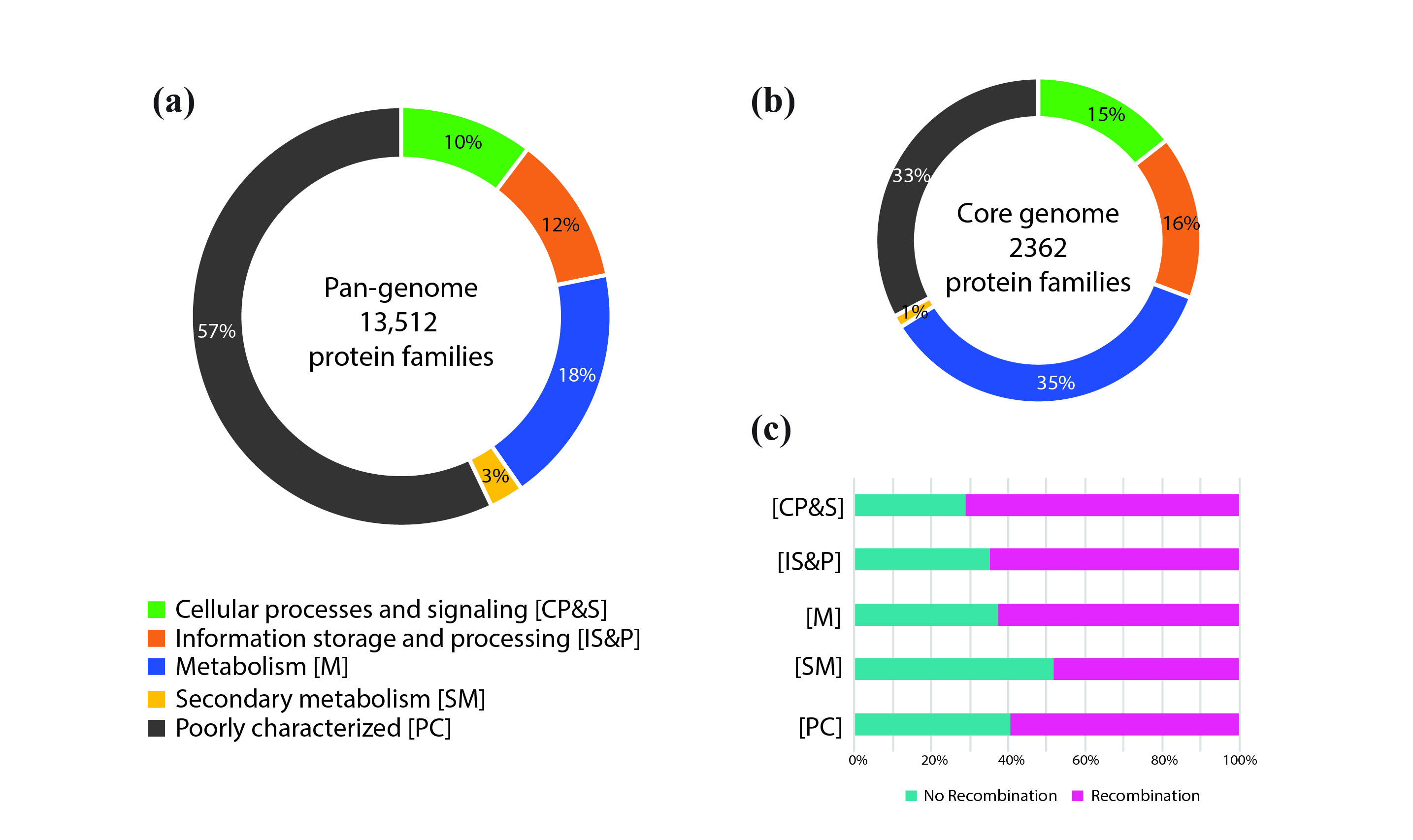


**Supplementary Figure S1.** Orthologous group distributions. **(a)** Pan-genome functional categories according to orthologous group assignments. **(b)** Core genome functional categories according to orthologous group assignments. **(c)** Proportion of core genome under recombination by functional category.

**
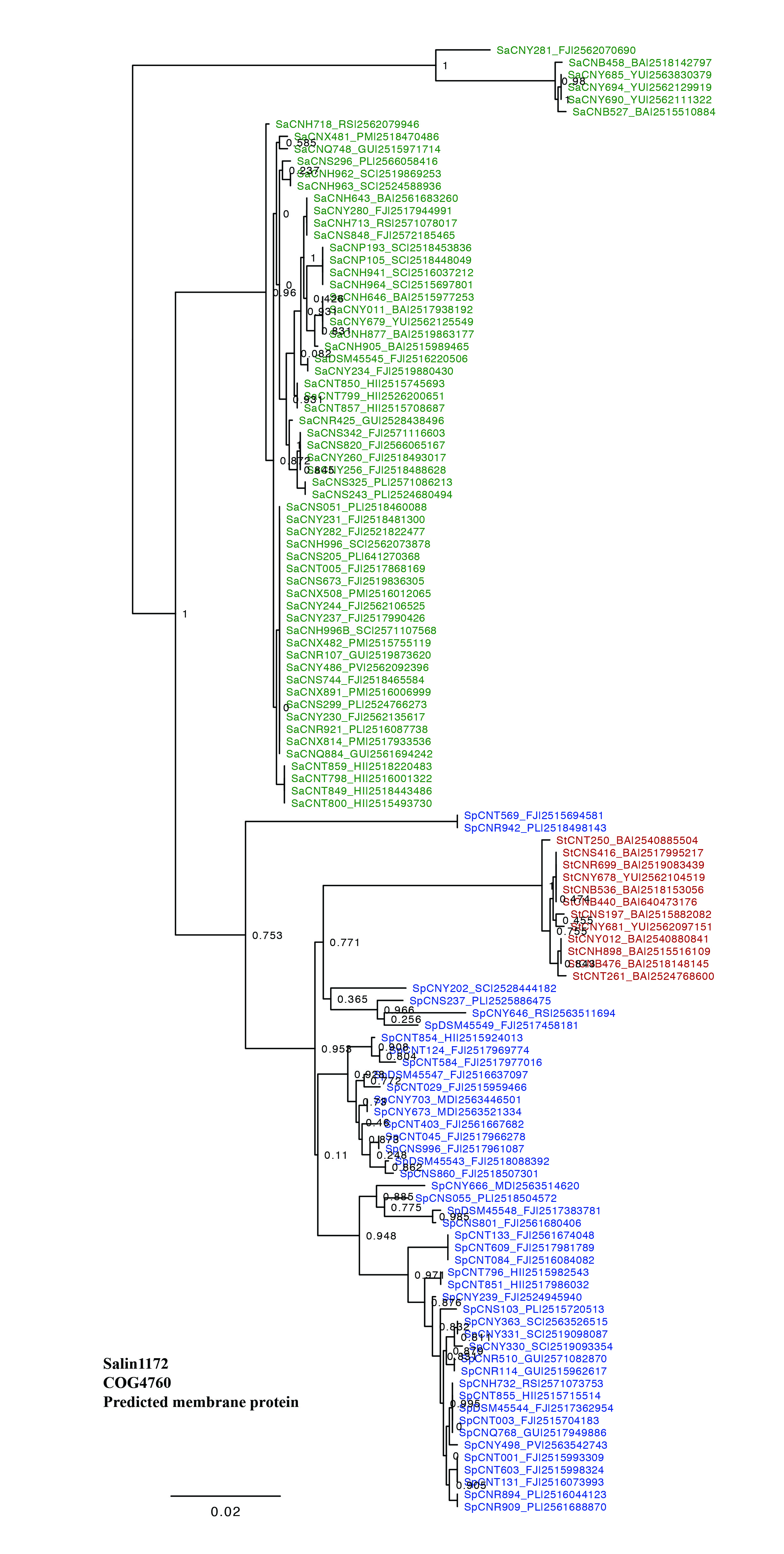
**

**Supplementary Figure S2.** Salin1172 represents an example of a gene in the core genome that generated a phylogeny that is incongruent with the single copy core gene phylogeny. Color-coded by *Salinispora* species: *S. tropica* (red), *S. arenicola* (green), *S. pacifica* (blue).


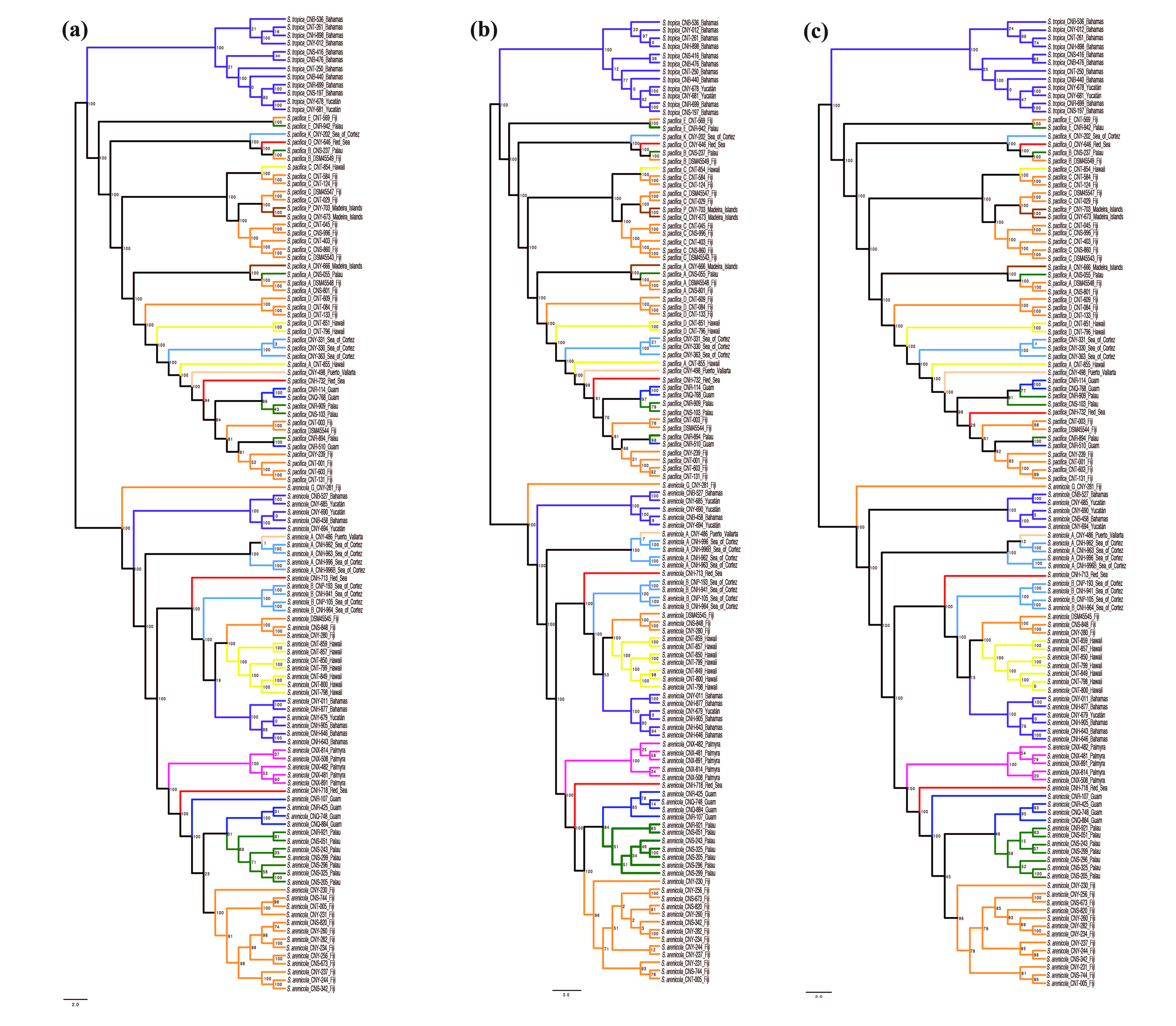


**Supplementary Figure S3.** Astral species phylogeny (cladogram) based on all shared single-copy genes (a), genes with no evidence of recombination (b) and genes with evidence of recombination (c). Species names are followed by strain identifier (starting with CN) and sampling location (purple: Bahamas and Yucatán, orange: Fiji, green: Palau, light blue: Sea of Cortez, red: Red Sea, yellow: Hawaii, brown: Madeira Islands, peach: Puerto Vallarta, dark blue: Guam, pink: Palmyra). Numbers at the nodes represent support from 1,000 bootstrap replicates.

**
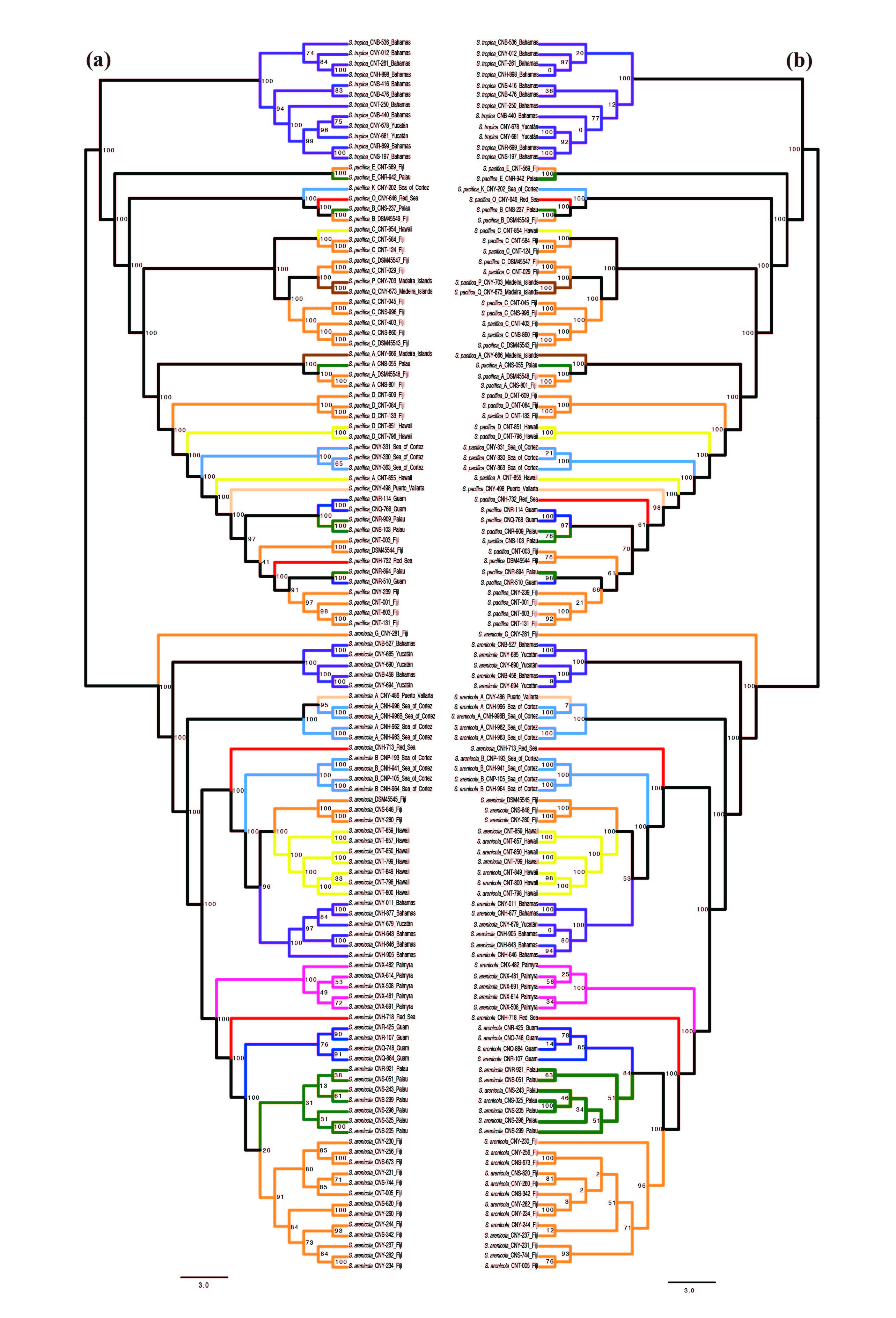
**

**Supplementary Figure S4.** Comparison of the concatenated tree using the genes not under recombination (a) and the ASTRAL species tree using the same data (b). Branches are transformed to cladogram view. Species names are followed by strain identifier (starting with CN) and the sampling location. Numbers on the branches represent the support from 1,000 (a) and 100 (b) bootstrap replicates. Color-coded by location (purple: Bahamas and Yucatán, orange: Fiji, green: Palau, light blue: Sea of Cortez, red: Red Sea, yellow: Hawaii, brown: Madeira Islands, peach: Puerto Vallarta, dark blue: Guam, pink: Palmyra).

**Supplementary Figure S5.** Linear regression of ANI vs. 16S rRNA nucleotide changes.


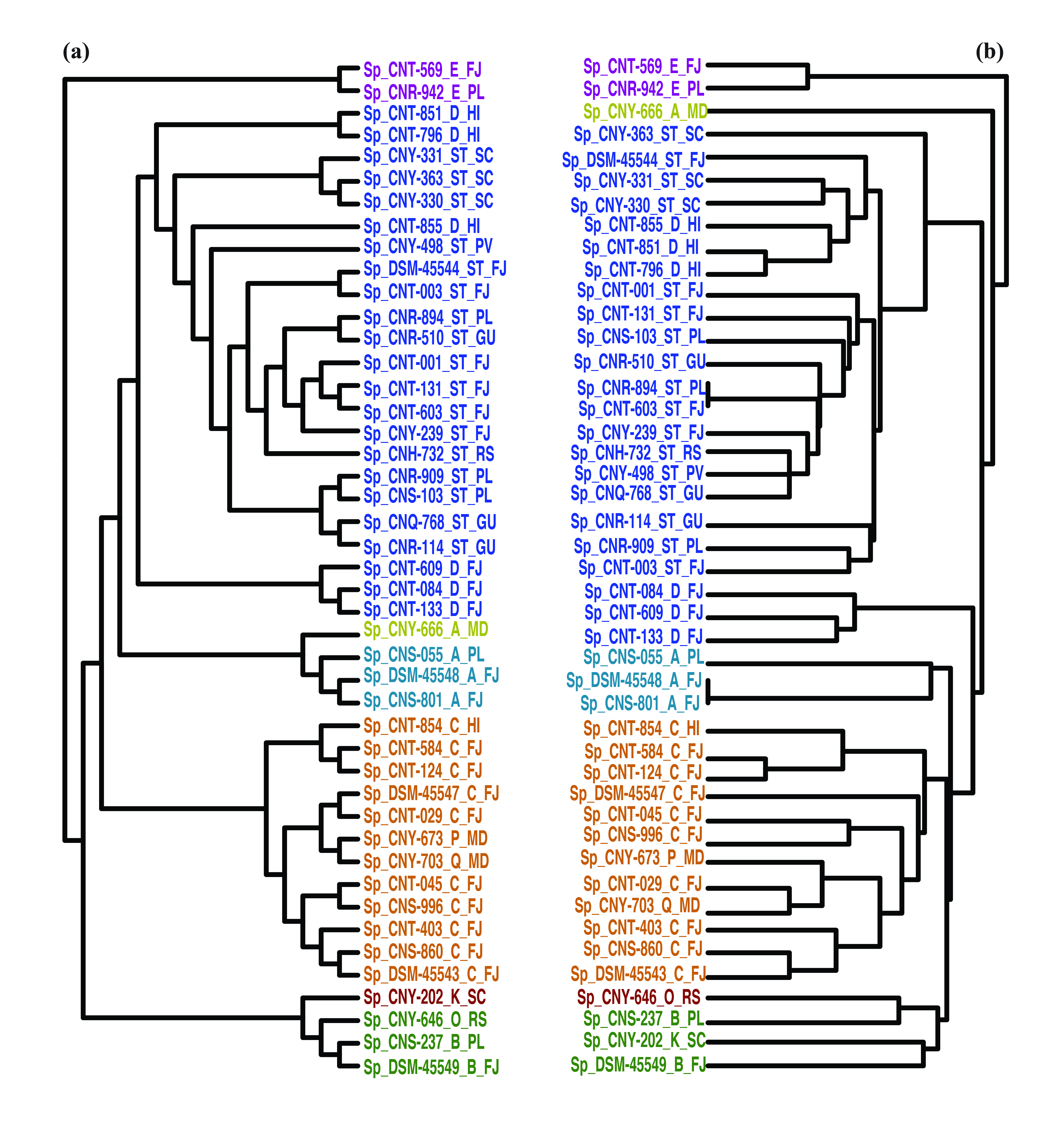


**Supplementary Figure S6.** Comparison of the *S. pacifica* single copy core gene phylogeny (a) with a hierarchical cluster analysis based on the presence/absence of secondary metabolite BGCs (b). Strains are color-coded based on 95% ANI groups. Strain identifiers: Sp = *S. pacifica*, followed by strain number, 16S rRNA sequence type (letter), and geographic origin (FJ = Fiji, GU = Guam, HI = Hawaii, MD = Madeira Islands, PL = Palau, RS = Red Sea, SC = Sea of Cortez).


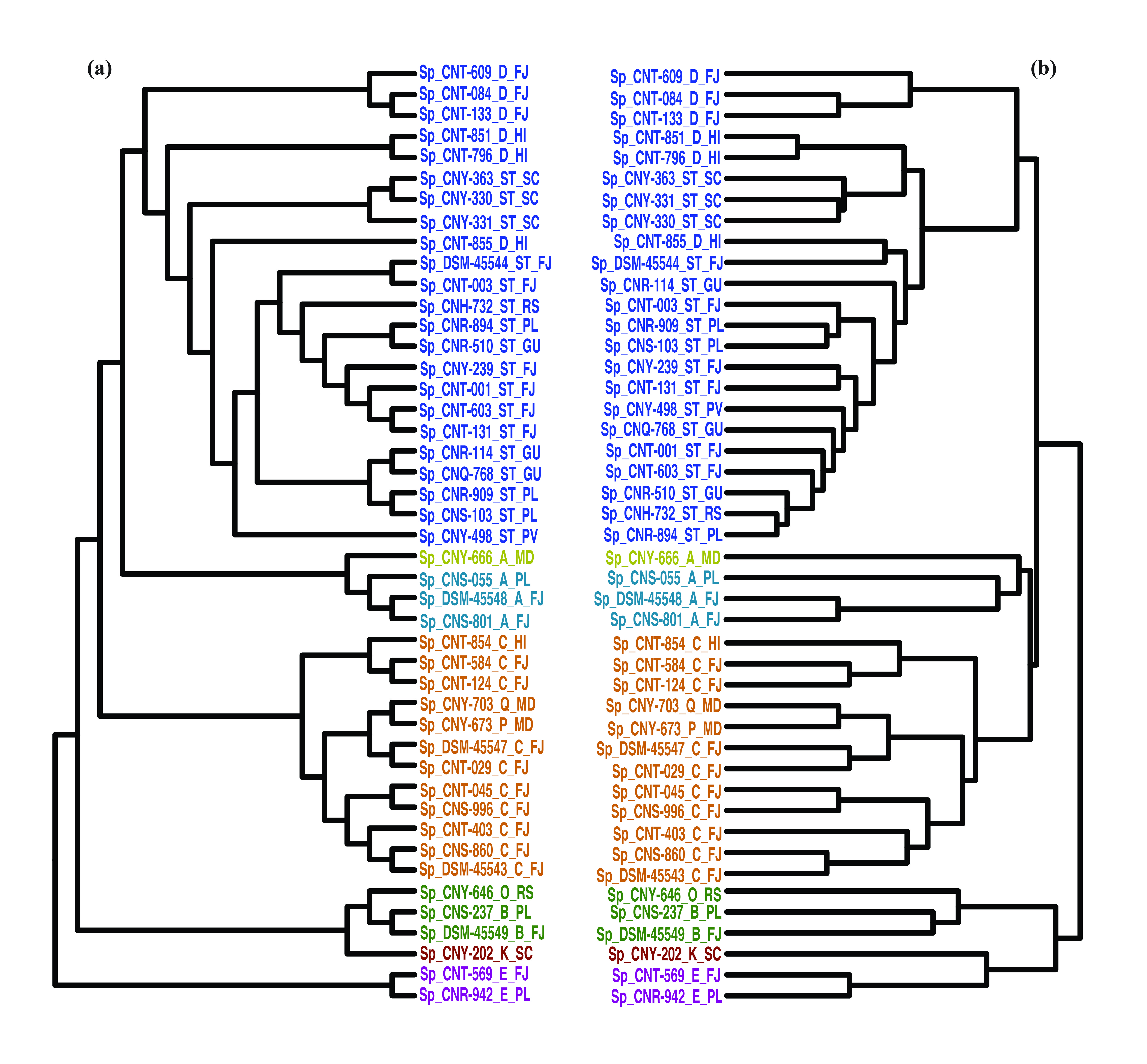


**Supplementary Figure S7.** Comparison of the *S. pacifica* single copy core gene phylogeny (a) with a hierarchical cluster analysis based on the presence/absence of OGs associated with COG category C: energy production and conversion (b). Strains are color-coded based on 95% ANI groups with the clade associated with the *S. pacifica* type strain (CNR-114) in blue. Strain identifiers: Sp = *S. pacifica*, followed by strain number, 16S rRNA sequence type (letter), and geographic origin (FJ = Fiji, GU = Guam, HI = Hawaii, MD = Madeira Islands, PL = Palau, RS = Red Sea, SC = Sea of Cortez).
